# Development and Population-Based Prognostic Utility Assessment of an ICD-10-CM-Based Prediction Model to Identify Left Ventricular Systolic Dysfunction: Discrimination Performance and Population-Based Evaluation in 83,357 Patients with Acute Coronary Syndrome

**DOI:** 10.3390/jcdd13100524

**Published:** 2026-10-17

**Authors:** Andreea Eunice Cosa, Marc Llagostera Martin, Sílvia Jovells-Vaqué, Mireia Andrés-Villareal, Emili Vela, Júlia Folguera Profitós, David Monterde, Jordi Piera-Jiménez, Gerard Carot-Sans, Raúl Ramos-Polo, Oona Meroño, Adrià Ricarte Marín, Natalia López Fernández, Albert Ariza-Solé, Cristina Enjuanes, Josep Comín-Colet

**Affiliations:** 1 Bio-Heart Cardiovascular Diseases Research Group, Bellvitge Biomedical Research Institute (IDIBELL), 08907 Barcelona, Spain; aeunice@idibell.cat (A.E.C.); sjovells@idibell.cat (S.J.-V.); mireia.andres.idi@gencat.cat (M.A.-V.); rramosp@bellvitgehospital.cat (R.R.-P.); cenjuanes@bellvitgehospital.cat (C.E.); 2 Centro de Investigación Biomédica en Red de Enfermedades Cardiovasculares (CIBERCV), Instituto Salud Carlos III, 28029 Madrid, Spain; 3 Cardiology Department, Heart Institute, Bellvitge University Hospital, 08907 Barcelona, Spain; mllagosteram@bellvitgehospital.cat (M.L.M.); omerono@bellvitgehospital.cat (O.M.); aariza@bellvitgehospital.cat (A.A.-S.); 4 Gerència d’Atenció Primària i Comunitària (GAPiC) Delta and Penedès, Catalan Health Institute, 08907 Barcelona, Spain; 5 Medicine Department, School of Medicine, International University of Catalonia, 08195 Barcelona, Spain; 6 Health Information System Department, Catalan Health Service, 08007 Barcelona, Spain; evela@catsalut.cat (E.V.); julia.folguera@catsalut.cat (J.F.P.); gerard.carot@catsalut.cat (G.C.-S.); 7 Digitalization for the Sustainability of the Healthcare System (DS3), 08006 Barcelona, Spain; dmonterde@gencat.cat (D.M.); jpiera@catsalut.cat (J.P.-J.); 8 Health Information System Department, Catalan Institute of Health, 08007 Barcelona, Spain; 9 Fundació TIC Salut i Social, 08009 Barcelona, Spain; 10 Community Heart Failure Program, Cardiology Department, Bellvitge University Hospital, 08907 Barcelona, Spain; 11 Functional Data Management Unit, Bellvitge University Hospital, 08907 Barcelona, Spain; aricarte@bellvitgehospital.cat (A.R.M.); natalialopez@bellvitgehospital.cat (N.L.F.); 12 Department of Clinical Sciences, School of Medicine, Universitat de Barcelona, 08907 Barcelona, Spain

**Keywords:** acute coronary syndrome, left ventricular ejection fraction, statistical learning, ICD-10-CM, heart failure, risk stratification

## Abstract

Population-based studies of acute coronary syndrome (ACS) are often limited by the lack of structured left ventricular ejection fraction (LVEF) data in administrative registries. We developed a logistic regression model using ICD-10-CM diagnostic codes and two clinical variables to estimate the probability of reduced LVEF (<50%) at discharge after ACS, and examined the prognostic value of the resulting classification in a large population-based cohort. The model was derived from 1596 patients with pre-discharge echocardiographic LVEF. In a 10-fold cross-validation analysis that repeated predictor selection within each train-ing fold, the procedure yielded a ROC-AUC of 0.711 (95% CI, 0.680–0.741). The fixed 28-predictor model, subsequently applied to the population-based cohort, yielded a ROC-AUC of 0.738 in the corresponding cross-validation analysis. The fixed 28-predictor model was then applied to 83,357 patients from the population-based BEAT-HF cohort (Catalonia, 2011–2021), in whom reference LVEF was unavailable. Patients classified as having reduced LVEF had a less favourable clinical profile and significantly higher rates of adverse cardiovascular outcomes. Predicted reduced LVEF was associated with higher observed rates of all-cause mortality (HR 2.45, 95% CI 2.35–2.55; *p* < 0.001), as well as higher risks of heart failure (HF) and major adverse cardiovascular events. The model should not guide individual clinical decisions but may support exploratory population-level characterization of patients in administrative data. Validation against measured LVEF is needed.

## 1. Introduction

Ischemic heart disease remains one of the leading causes of morbidity, mortality, and disability-adjusted life years lost worldwide [1,2]. In this context, acute coronary syndrome (ACS) represents a medical emergency with high global prevalence, substantial healthcare resource utilization, and significant economic burden [3]. Moreover, ACS exerts a direct impact on both life expectancy and quality of life [4,5]. Consequently, research focused on the prevention and early diagnosis of ischemic heart disease, as well as on the identification of prognostic factors that enable accurate risk stratification, is of paramount importance.

Left ventricular ejection fraction (LVEF) following an ACS event is a key parameter for both risk stratification and prognostic assessment in affected patients [6]. Multiple studies have established that reduced LVEF is associated with higher long-term mortality, significantly increasing the risk of heart failure (HF), rehospitalization, and major adverse cardiovascular events (MACE) [6,7,8,9]. Conversely, improvement in LVEF after intervention or during follow-up is associated with reduced mortality and enhanced functional outcomes [10].

In clinical and epidemiological research, accurate diagnostic coding using standardized diagnostic coding systems such as the International Classification of Diseases, 10th revision, Clinical Modification (ICD-10-CM), constitutes an essential tool for reliable medical data recording, population surveillance, and scientific knowledge generation. In the field of ischemic heart disease, the use of these codes enables precise identification of ACS cases within administrative databases and electronic health record repositories, thereby facilitating the development of observational studies and the monitoring of health outcomes [11,12,13]. However, these coding systems present relevant limitations: LVEF is not directly codified as an independent entity and must instead be inferred through subtypes of HF (systolic or diastolic) or associated procedures. This constraint reduces the ability to differentiate between distinct post-ACS risk profiles and limits the development of population-based studies aimed at evaluating the prognostic impact of LVEF.

Hence, the primary objective of this study was to develop a logistic regression prediction model to estimate the probability of reduced LVEF (<50%) at discharge in post-ACS patients, using standardized diagnostic codes and routinely available clinical information. The secondary objective was to assess the prognostic utility of the model-derived classification, that is, its ability to stratify the risk of adverse outcomes, in a real-world population-based cohort in which LVEF was unknown. This secondary analysis was not designed to validate the diagnostic accuracy of the model.

## 2. Materials and Methods

### 2.1. Data Sources and Population

The objectives of this observational, retrospective study were [1] to develop a logistic regression prediction model to estimate the probability of left ventricular systolic dysfunction (LVEF < 50%) at discharge after ACS, and [2] to assess the prognostic utility of the model-derived classification (i.e., its association with adverse clinical events) in a real-world population-based cohort in which LVEF was unavailable. The prediction model was developed using data from a local registry of consecutive patients discharged after an ACS between 1 January 2009, and 31 December 2010, managed at the University Hospital of Bellvitge (L’Hospitalet de Llobregat, 08907 Barcelona, Spain). The local registry, which contained clinical data (including key cardiovascular features), was enriched with a claims registry containing life-long cumulative diagnostics. Patients without myocardial infarction (MI) were excluded because MI is the main determinant of acute left ventricular systolic dysfunction in ACS, while non-infarction presentations differ substantially in both mechanism and risk. Patients with pre-existing HF were excluded to avoid confounding LVEF prediction with prior ventricular dysfunction.

The prognostic utility of the model was assessed on a population-based database of individuals discharged after an ACS between 1 January 2011 and 31 December 2021 from any of the 68 hospitals providing universal healthcare under the Catalan Health System. This database was built in the context of the BEAT-HF study (clinicaltrials.gov, identifier NCT06255418) [11]. Briefly, the BEAT-HF study was an epidemiological analysis of incident HF among individuals with hospital discharge after an ACS episode and their clinical and resource burden. Data for this study was gathered from the Catalan Health Surveillance System, which systematically collects diagnostics, medicines, relevant clinical outcomes (e.g., mortality, non-planned hospitalizations, and admissions to emergency room) and resource use (e.g., primary care visits, specialized visits, and admissions to hospital and intermediate care resources) from all hospitals, primary care, mental health and intermediate care centres providing universal care to the Catalan population [14].

The study was approved by the Clinical Research Ethics Committee of the Bellvitge Biomedical Research Institute, Spain, and conducted in accordance with the Declaration of Helsinki. Given the retrospective nature of the study and the use of pseudonymised data, the need for written informed consent was waived.

### 2.2. Data Preparation

The local registry (with clinical and cardiovascular features) and the administrative claims database (with life-long cumulative diagnostics), used for model development, were linked using a key table containing the internal study identifier and the corresponding unique healthcare identifier assigned by the Catalan Health Service. Following linkage, the resulting dataset underwent data cleaning procedures, including the removal of records with inconsistent identifiers, erroneous entries, duplicate records, and implausible values identified during quality control checks.

Diagnoses in the claims database were originally coded using the International Classification of Diseases, Ninth Revision, Clinical Modification (ICD-9-CM). To ensure compatibility with the population-based prognostic evaluation dataset, which used the International Classification of Diseases, Tenth Revision, Clinical Modification (ICD-10-CM), all ICD-9-CM diagnosis codes were mapped to their corresponding ICD-10-CM codes prior to model development.

Categorical predictor variables were transformed using one-hot encoding, generating binary indicator variables for each category. This approach enabled the inclusion of categorical information in algorithms requiring numerical input.

For the prognostic utility assessment, the population-based cohort was further refined to include only individuals with a valid healthcare identifier and an index ACS episode. Inter-hospital transfers and transfers outside Catalonia were excluded to avoid duplicate episode registration and incomplete follow-up. Individuals with a history of HF before the index ACS admission were also excluded, as the objective was to assess the prognostic utility of model-predicted LVEF in patients without established HF at baseline. Follow-up data were then used to identify incident HF and other adverse clinical outcomes.

### 2.3. Outcomes and Predictors

*Prediction time point and predictor window*: The prediction time point was hospital discharge after the index ACS admission. The model is therefore a discharge-time statistical classification model; it was not designed as an admission-time model or an early-triage tool. Candidate diagnostic predictors were derived from each patient’s longitudinal administrative record in the Catalan Health Surveillance System, which stores all diagnostic codes recorded for a person. The predictor window comprised all diagnostic information available up to and including the discharge date of the index ACS hospitalization, that is, historical diagnoses recorded before admission and diagnoses documented during the index hospitalization. Diagnoses first recorded after discharge, post-discharge follow-up events and subsequent clinical outcomes were not used as predictors.

The local registry of patients discharged alive after an ACS episode included sex, admission date, LVEF, age, MI location, Killip class, smoking status, diabetes mellitus, hypertension, dyslipidaemia, peripheral artery disease, chronic kidney disease, prior coronary artery bypass graft, previous atrial fibrillation, previous HF, chronic obstructive pulmonary disease, anaemia, ST-elevation MI, and prior stroke. To these key cardiovascular features, we added all diagnostic codes available in the longitudinal administrative record within the prespecified predictor window.

*Outcome*: The model was developed to estimate the probability of left ventricular systolic dysfunction (defined as LVEF < 50%). To assess its prognostic utility in a real-world setting, the model was applied to the evaluation cohort and patients were classified according to their model-predicted LVEF as predicted preserved LVEF (ppLVEF; ≥50%) or predicted reduced LVEF (prLVEF; <50%). The prognostic analysis assessed associations between these model-defined groups and adverse clinical outcomes; it did not assess diagnostic accuracy in the population-based cohort, where reference LVEF measurements were unavailable. The following adverse outcomes were investigated in the two groups: all-cause mortality, recurrent MI or ACS, stroke or transient ischemic attack (TIA), incident HF, heart transplantation, and mechanical circulatory support implantation. The 4-point MACE (major adverse cardiovascular events) composite endpoint was defined as a composite of all-cause mortality, recurrent ACS, new coronary revascularization, and stroke/TIA [11].

*Predictor selection*: Candidate predictors were initially explored through descriptive analyses. To reduce the dimensionality of the predictor set and limit the risk of overfitting, univariable Chi-square (χ^2^) tests were applied using a significance threshold of *p* < 0.05. A Pearson correlation analysis and multicollinearity assessment were then conducted by calculating Variance Inflation Factors (VIFs) for the selected predictors.

### 2.4. Model Training and Evaluation

A logistic regression model was used for predictive development, as the goal was to obtain a binary response (LVEF < 50% or not) [15,16].

To assess model generalization and prevent overfitting, stratified 10-fold cross-validation (CV) was applied. The dataset was divided into 10 folds of similar size while preserving class proportions. For each iteration, the model was trained on 9 folds and evaluated on the remaining fold. Within each training fold, predictor selection was repeated using the same development pipeline described above, including univariable χ^2^ screening, correlation assessment, and VIF diagnostics. This approach ensured that predictor selection was performed independently within each training fold before model fitting. Held-out predicted probabilities were retained for all participants, and the resulting pooled out-of-fold predictions were used to obtain the primary estimate of model performance [17,18].

Model discrimination and classification performance were assessed using accuracy, sensitivity (recall), specificity, and precision. The F1-score was also calculated. The receiver operating characteristic (ROC) and the area under the ROC curve (AUC) were also estimated [19].

A fixed predictor specification was obtained by performing predictor selection once in the full derivation cohort before cross-validation. This fixed predictor set was then evaluated using the same 10-fold cross-validation folds and was subsequently applied without refitting to the population-based BEAT-HF cohort. Performance was summarized across the 10 iterations using the mean and standard deviation of the evaluation metrics. This evaluation describes the performance of the final predictor set that was subsequently applied to the population-based BEAT-HF cohort. To complement the original fold-level cross-validation summaries, a held-out predicted probability was retained for each patient across the stratified 10-fold CV procedure. These pooled out-of-fold predictions were used to estimate the Brier score, calibration-in-the-large, calibration slope, precision–recall AUC, and threshold-dependent performance measures at the original probability threshold of 0.50. Ninety-five percent confidence intervals were estimated using 2000 non-parametric bootstrap resamples of paired outcomes and out-of-fold predicted probabilities.

Finally, to enhance model interpretability, the SHAP (Shapley Additive Explanations) method was applied. SHAP increases model transparency in scenarios where interpretability is critical, such as clinical settings. It allows researchers to understand why the model makes certain predictions, which variables are most influential, and how their contributions vary across cases [20].

The model development, evaluation and interpretability analyses were conducted in Python (version 3.11.3; Python Software Foundation, Wilmington, DE, USA) with scikit-learn, pandas, and NumPy libraries.

### 2.5. Analytical Methods

Baseline characteristics were presented as frequencies and percentages for categorical variables and as mean and the standard deviation (SD) for continuous variables. Group comparisons used Chi-square (χ^2^) or Fisher exact tests for categorical variables and Student’s *t*-test or U Mann–Whitney for continuous variables, as appropriate.

In the evaluation cohort, patients were stratified into ppLVEF (≥50%) and prLVEF (<50%) based on model-predicted LVEF. Clinical outcomes were analysed using time-to-event methods. Kaplan–Meier survival curves were constructed for main events, with group differences assessed using the log-rank test. Incidence rates were calculated per exposure group. Cox proportional hazards regression models were used to estimate HRs and their corresponding 95% confidence intervals (95% CIs) for all-cause mortality associated with prLVEF. For all other adverse outcomes, including recurrent MI, stroke/transient ischemic attack, MACE, heart transplantation, and MCS implantation, subdistribution hazard ratios (sHRs) were estimated using Fine and Gray competing-risk regression models, considering death as a competing event. Event occurrence probabilities at 2, 5, and 10 years were estimated according to exposure group [21].

Because reference LVEF was not available in the prognostic evaluation cohort, these analyses describe the association between model-derived groups and outcomes; they do not evaluate the discrimination, calibration or diagnostic accuracy of the model in that cohort.

All statistical tests were two-sided, with *p* < 0.05 considered statistically significant. Statistical analyses were performed using R software (version 4.0.2; R Foundation for Statistical Computing, Vienna, Austria).

## 3. Results

### 3.1. Baseline Characteristics of Model Development Cohort and Variable Selection

**Figure 1:**
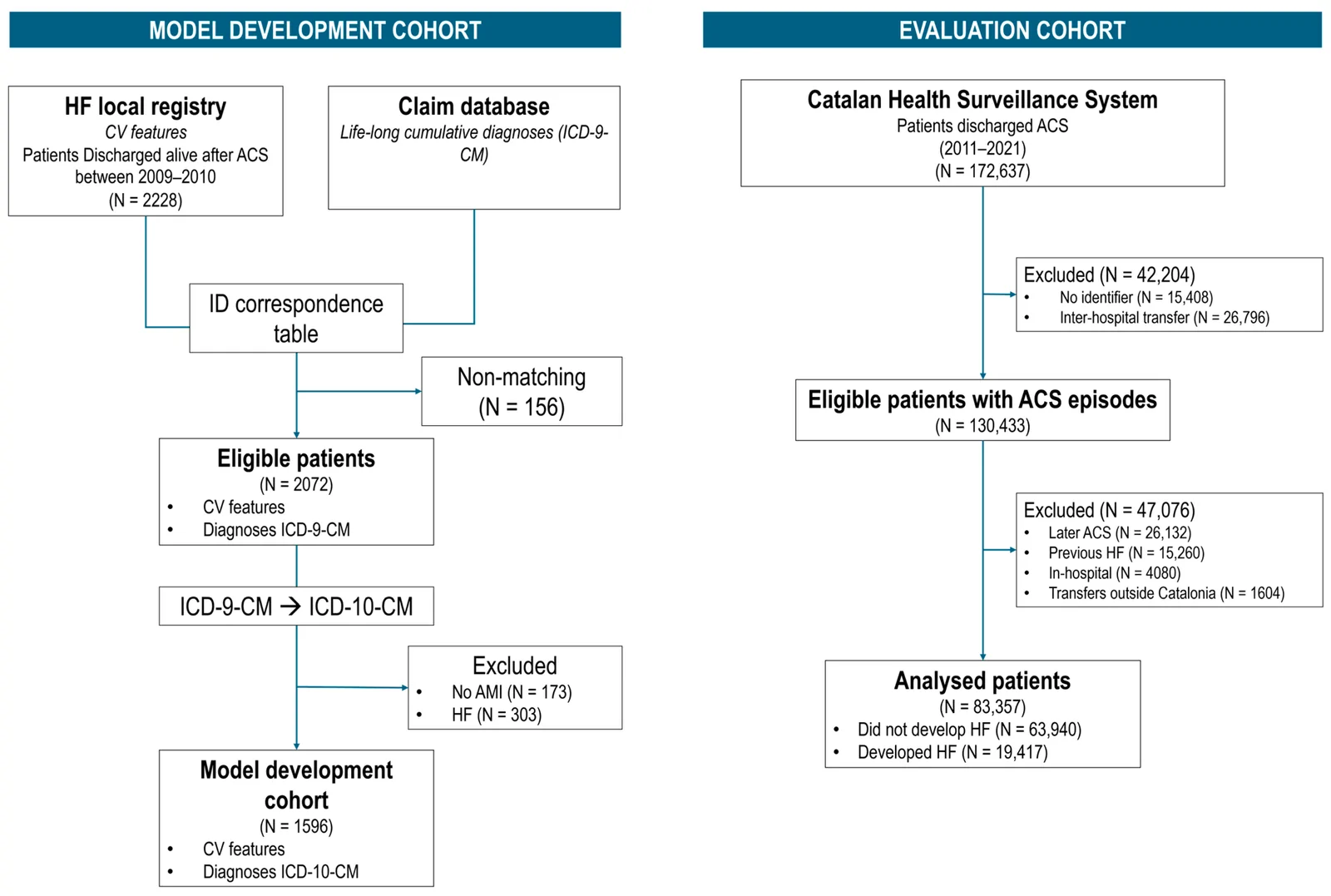
Flow chart of patient selection for the model development cohort and the prognostic evaluation cohort, aimed at assessing the prognostic utility of the model. CV: Cardiovascular; HF: Heart Failure, ACS: Acute Coronary Syndrome: AMI: Acute Myocardial Infarction.

Figure 1 summarizes the patient selection process for the derivation and population-based prognostic evaluation cohorts. The local registry included 2228 patients discharged alive after an ACS episode between 2009 and 2010. Of these, 2072 patients were successfully linked to the Catalan Health Surveillance System and had available clinical and diagnostic-code information; 156 patients could not be linked and were excluded. Among the 2072 linked patients, 173 were excluded because no myocardial infarction was documented and 303 were excluded because of HF according to the prespecified administrative definition. The resulting derivation cohort comprised 1596 patients with available echocardiographic LVEF and ICD-10-CM diagnostic information.

Patients in the derivation cohort were classified according to the reference echocardiographic measurement as having preserved LVEF (LVEF ≥ 50%) or reduced LVEF (LVEF < 50%). Of the 1596 patients, 1142 (71.6%) had preserved LVEF and 454 (28.4%) had reduced LVEF. The proportion of women was similar between groups (20.9% vs. 19.6%; *p* = 0.555). Patients with reduced LVEF were older than those with preserved LVEF (mean (SD)), 63.12 (13.0) vs. 61.12 (12.2) years; *p* = 0.005) and were more frequently aged >75 years (20.3% vs. 14.0%; *p* = 0.002). They also had a higher frequency of anterior MI (59.9% vs. 34.7%; *p* < 0.001), whereas inferior MI was less frequent (26.2% vs. 50.9%; *p* < 0.001). Patients with reduced LVEF had a more severe clinical presentation, with Killip class > I in 26.7% compared with 5.7% among those with preserved LVEF (*p* < 0.001). Diabetes mellitus, previous MI, previous atrial fibrillation, anaemia, STEMI, and previous stroke were more frequent in the reduced-LVEF group. Baseline characteristics are presented in Table 1. The 30 most frequent ICD-10-CM diagnostic codes among the 1596 patients in the derivation cohort are summarized in Table S1.

The candidate predictor set comprised 827 variables, including diagnostic-code indicators and clinical variables available within the prespecified predictor window. For model development, not all candidate variables were used. The χ^2^ test performed using a significance threshold of *p* < 0.05 yielded 28 statistically significant variables: 26 of them ICD-10-CM diagnostic codes and 2 clinical features. The complete list of selected variables, along with their ICD-10-CM codes and descriptions, is provided in Table 2.

**Figure 2:**
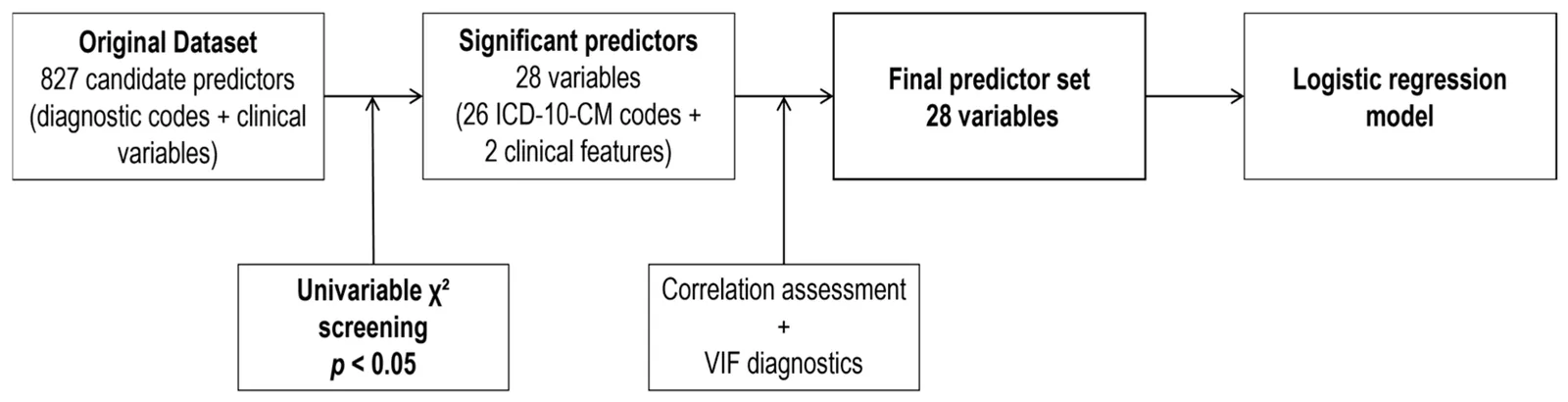
Flow chart of variables selection for the logistic regression-based model.

**Figure 3:**
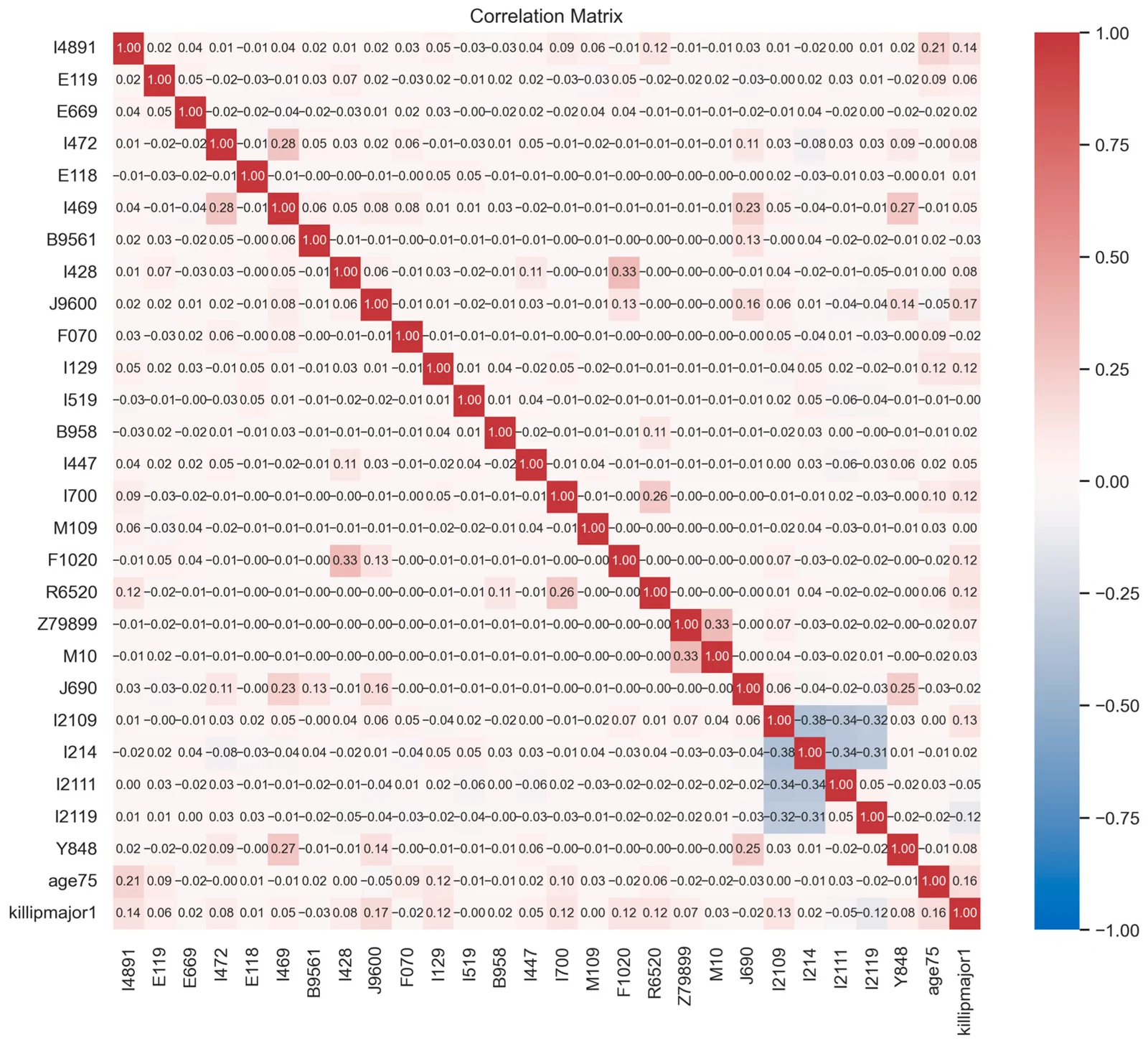
Correlation matrix of the variables to be introduced in the model.

The correlation analysis performed on the 28 retained predictors showed no evidence of strong pairwise linear relationships (Figure 2). The resulting heatmap displays the Pearson correlation coefficients between all pairs of predictors, with values ranging from −1.0 to 1.0 (Figure 3).

The collinearity analysis performed on the 28 candidate variables revealed minimal correlation among predictors, with VIF values ranging from 1.01 to 1.33 (Table S2). These results confirm the absence of significant multicollinearity and support the stability of the regression estimates.

### 3.2. Internal Performance of the Model in the Derivation Cohort

*Cross-validation including predictor selection within each training fold:* The primary estimate of model performance was obtained using a stratified 10-fold cross-validation procedure in which predictor screening, correlation assessment and VIF diagnostics were repeated independently within each training fold. The number of diagnostic codes retained from the 827 set varied between 36 and 46 across folds, and 19 diagnostic codes plus age > 75 years and Killip class > I were selected in all 10 folds (Table S3). In this analysis, the pooled out-of-fold ROC-AUC was 0.711 (95% CI 0.680–0.741), accuracy was 0.756 (0.735–0.776), sensitivity 0.289 (0.248–0.330) and specificity 0.941 (0.928–0.955); the Brier score was 0.172 (0.161–0.183), calibration-in-the-large −0.097 (−0.244 to 0.066) and the calibration slope 0.874 (0.749–1.005) (Tables S4 and S5, Figure S1).

**Figure 4:**
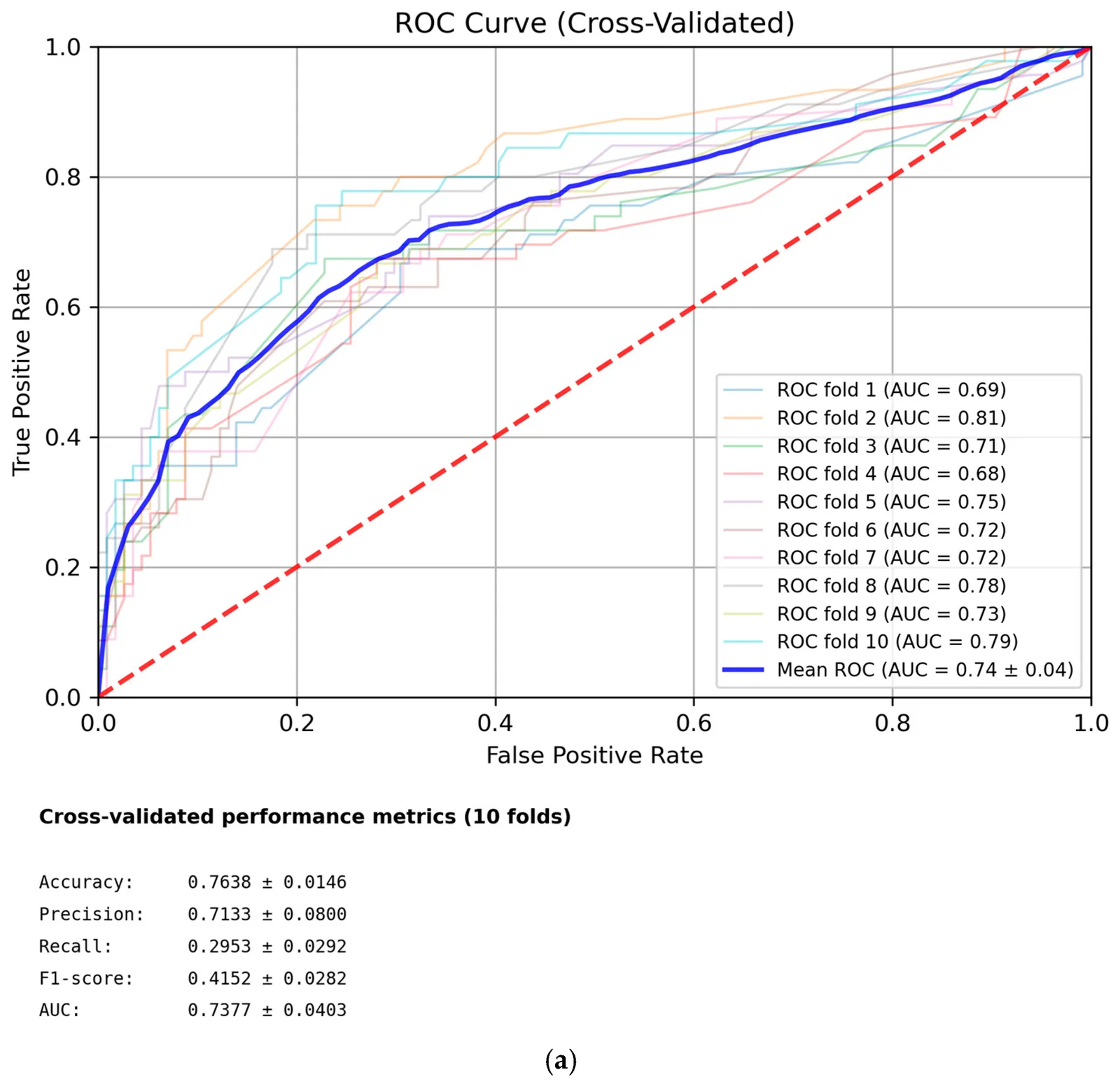

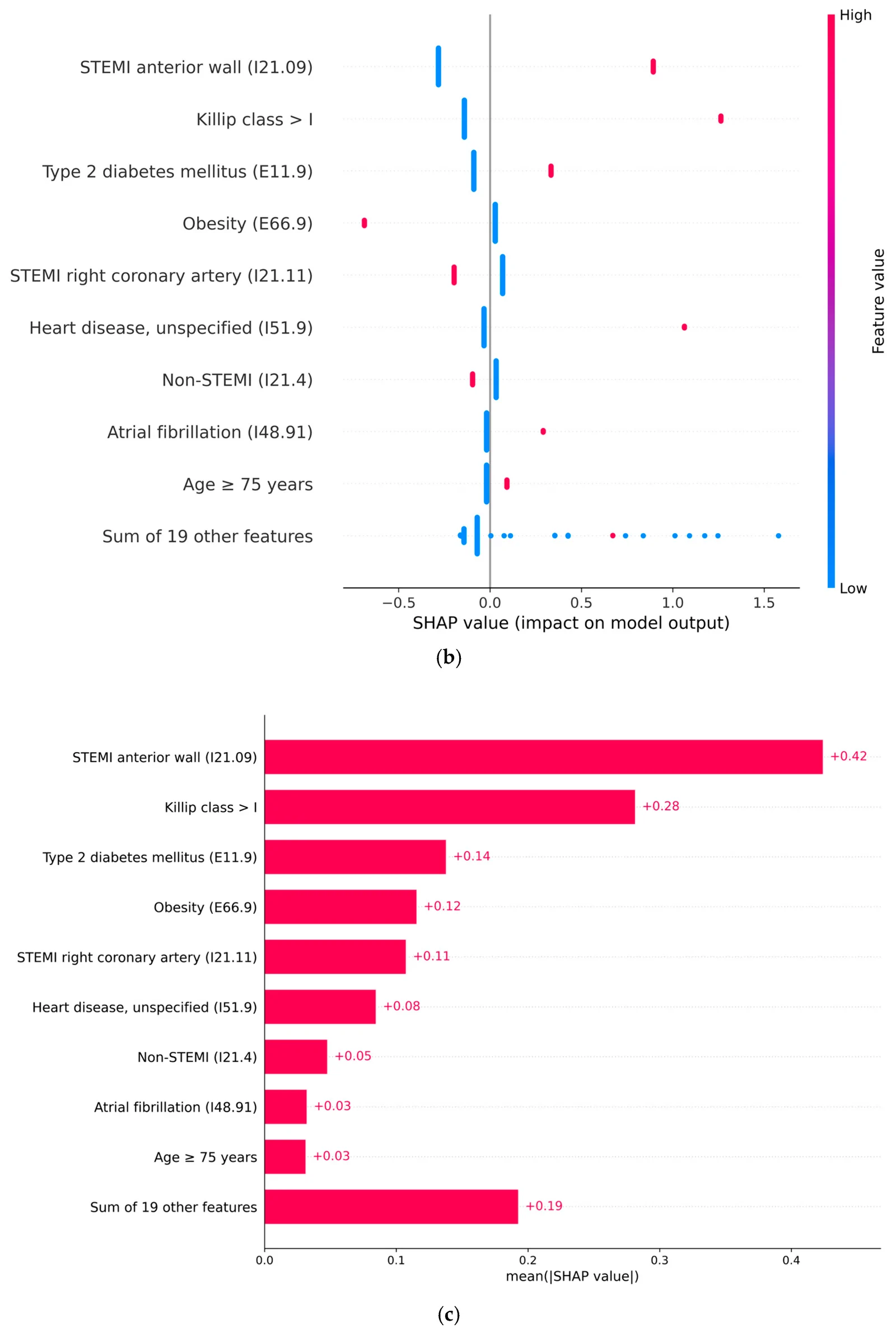
(**a**) ROC curves and scores. The red dashed diagonal line represents the performance of a non-discriminative classifier (AUC = 0.50), equivalent to random prediction. (**b**) SHAP values to explain the predicted odds of LVEF < 50%. STEMI: ST-elevation myocardial infarction. (**c**) Importance of the SHAP characteristic measured as Shapley absolute mean values. I2109 was the most important characteristic.

*Fixed-predictor model:* The model fitted with the fixed 28 predictors was evaluated using the same stratified 10-fold cross-validation framework. This analysis yielded a mean accuracy of 0.764 (0.015), precision of 0.713 (0.080), recall of 0.295 (0.029), and F1-score of 0.415 (0.028). The average ROC-AUC was 0.738 (0.040), indicating moderate and fair discriminative ability to distinguish patients with LVEF < 50% (Figure 4a). This analysis describes the performance of the final predictor set that was ultimately applied to the population-based BEAT-HF cohort.

While some variability exists between folds (e.g., fold 1 or 4 with AUC 0.69 and 0.68), the overall performance remains consistently better than random classification. These results suggest that the selected features carry relevant predictive information and warrant external validation in an independent cohort with reference LVEF measurements.

The mean absolute SHAP values further confirmed the dominance of two features, with I21.09 (ST elevation MI (STEMI) of anterior wall) contributing an average impact of +0.41 and Killip–Kimball classification value of +0.31, followed by E66.9 (obesity), E11.9 (Type 2 diabetes mellitus), I21.11 (STEMI involving the right coronary artery), and others with smaller but relevant impacts. A group of 19 additional variables contributes a combined effect of +0.15, suggesting that the model integrates multiple lower-weighted clinical and diagnostic factors (Figure 4b,c).

*Pooled out-of-fold assessment and calibration of the fixed-predictor model*: In the additional pooled out-of-fold predictions from the fixed 28-predictor model (*n* = 1596; observed prevalence of LVEF <50%, 28.4%), the ROC-AUC was 0.732 (95% CI 0.702–0.761) and the precision–recall AUC 0.578 (0.532–0.627). The Brier score was 0.166 (0.155–0.177), lower than that of the null model (0.204). Calibration-in-the-large was −0.010 (−0.181 to 0.158) and the calibration slope 0.976 (0.852–1.111), close to their ideal values of 0 and 1. At the 0.50 threshold, sensitivity was 29.5% (25.4–33.7%) and specificity 95.2% (93.9–96.4%). Of the 454 patients with observed LVEF <50%, 134 were classified as positive and 320 as negative; thus, approximately 70% of patients with reduced LVEF were missed at this threshold (Table S6).

**Figure 5:**
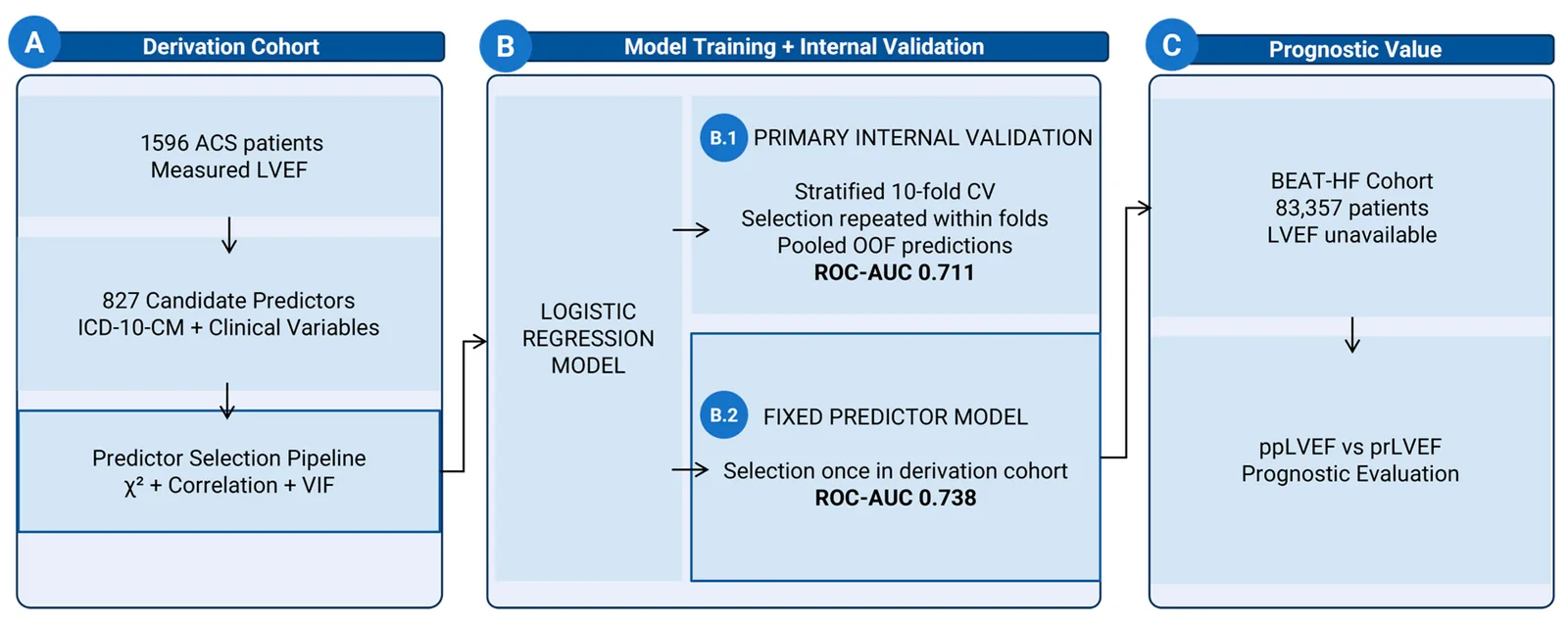
Overview of model development, internal validation, and prognostic evaluation.

An overview of the derivation cohort, model development, internal validation, and subsequent prognostic evaluation is shown in Figure 5.

The derivation cohort included 1596 patients with ACS and measured LVEF. Candidate predictors were subjected to the predefined predictor selection pipeline. The primary internal validation used leakage-aware stratified 10-fold cross-validation with predictor selection repeated within each training fold, yielding a ROC-AUC of 0.711. A secondary fixed-predictor analysis yielded a ROC-AUC of 0.738. The final predictor set was subsequently applied to the 83,357-patient BEAT-HF cohort, in which measured LVEF was unavailable, for prognostic evaluation.

### 3.3. Baseline Characteristics of Evaluation Cohort (BEAT-HF Cohort)

83,357 patients without previous HF were discharged after ACS between 1 January 2011 and 31 December 2021. Reference LVEF was not available for these patients. The mean age of this overall population was 66.8 years old (SD 13.4) and 71.4% (*N* = 59,543) were men. The patient selection of the study is represented in Figure 1 and all baseline characteristics are shown in Table 3.

After applying the LVEF prediction model in this cohort, patients were classified into two groups based on their predicted LVEF: ppLVEF and prLVEF. Baseline characteristics of both groups are likewise presented in Table 3. From the overall BEAT-HF cohort, 76,721 patients (92%) were classified into the ppLVEF group, while the remaining 8% were assigned to the prLVEF group.

The prLVEF group had a higher proportion of female patients (32.1% vs. 28.3%; *p* < 0.001) and was older (72.7 [13] vs. 66.3 [13.2] years; *p* < 0.001). Additionally, prLVEF group had a worse clinical profile compared to the ppLVEF group, showing higher burden of comorbidities including hypertension, type 2 diabetes mellitus, and chronic kidney disease. prLVEF ACS presentation was also notably more severe, with 58.1% presenting with Killip class > 1 (15.8% Killip class IV), compared to 5.59% in the ppLVEF group (0.38% Killip class IV).

### 3.4. Clinical Outcomes According to Model-Predicted LVEF Group

Patients classified as prLVEF exhibited a worse overall prognosis, with a significantly higher risk of adverse such as all-cause mortality (HR: 2.45; 95% CI: 2.35–2.55), recurrent MI (HR: 1.28; 95% CI: 1.19–1.37), stroke or transient ischemic attack (HR: 1.29; 95% CI: 1.16–1.43), and 4-points MACE (HR: 1.64; 95% CI: 1.59–1.70). These differences were even more pronounced for the incidence of terminal events, such as the need for heart transplantation (HR: 8.70; 95% CI: 5.33–14.20) or mechanical circulatory support implant (HR: 4.20; 95% CI: 2.43–7.26). All clinical outcomes are shown in Table 4.

**Figure 6:**
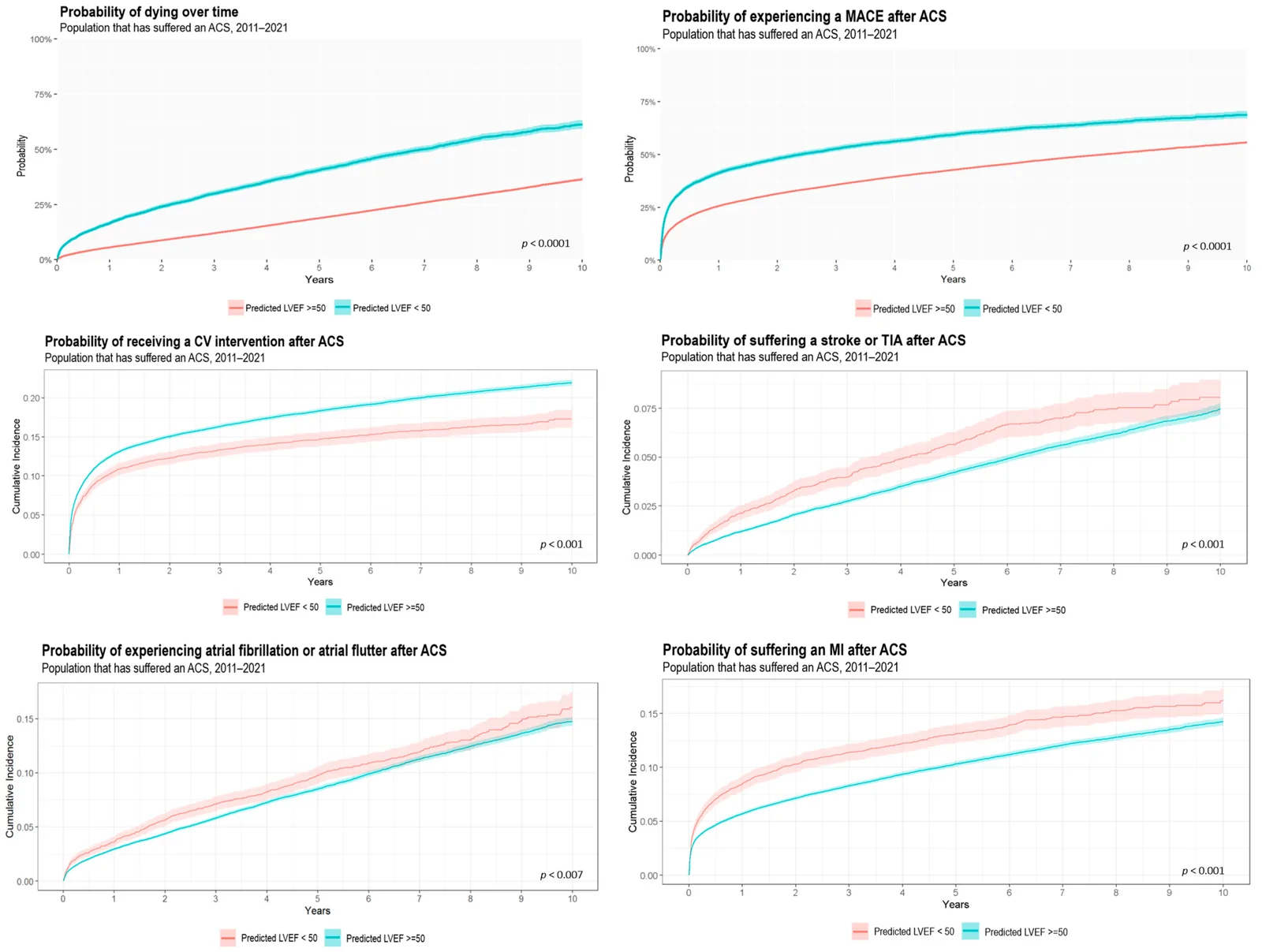
Time-to-event analyses according to predicted LVEF group (ppLVEF ≥ 50% vs. prLVEF < 50%). Kaplan–Meier curves were used to estimate all-cause mortality, with differences assessed using the log-rank test. For non-fatal outcomes, cumulative incidence functions were estimated using competing-risk methods, considering death as a competing event. Shaded areas represent 95% confidence intervals.

The adverse event incidence curves shown in Figure 6 illustrate clear differences in cumulative MACE risk between groups. Additionally, an early divergence in the curves following the index ACS event is evident. Consistent with these findings, the two-, five-, and ten-year probabilities of experiencing an adverse event were significantly higher in the prLVEF group compared to the ppLVEF group (Table 5).

## 4. Discussion

In this retrospective observational study, we developed a logistic regression prediction model to estimate the probability of LVEF < 50% using ICD-10-CM diagnostic codes in patients discharged alive after ACS and examined its ability to stratify risk in a population-wide administrative database with unknown LVEF. The model was derived from a single-centre cohort of 1596 patients and subsequently applied to a population-based cohort of 83,357 patients from the BEAT-HF study. In the derivation cohort, the primary estimate of model performance was obtained using cross-validation procedure in which predictor selection was repeated independently within each training fold, yielding a ROC-AUC of 0.711. The 28 fixed-predictor specification subsequently applied to the population-based cohort achieved a ROC-AUC of 0.738. In the population-based cohort, patients classified by the fixed-predictor model as having prLVEF exhibited a markedly worse prognosis, with a 2.45-fold higher risk of all-cause mortality and a 1.64-fold higher risk of 4-point MACE compared to those with ppLVEF. These findings indicate that diagnostic-code information can be used to classify ACS patients according to their model-estimated probability of reduced LVEF when reference echocardiographic data are unavailable.

LVEF is a fundamental parameter in the management of patients with ACS and HF. This parameter, which estimates the contractile capacity of the left ventricle, allows patients to be classified into two major groups based on the presence or absence of contractile dysfunction (preserved or reduced LVEF). This classification has a direct impact on patient management, prognosis assessment, and the selection of optimal pharmacological and device-based therapies [22]. However, several studies have shown that this parameter is frequently unknown, either because it has not been measured or properly recorded, with up to 29% of missing values reported [23,24,25]. This clearly limits the interpretation and real-world applicability of such studies.

This study presents the development and population-based prognostic evaluation of a logistic regression–based prediction model for reduced LVEF in patients discharged alive after ACS. The model was developed using electronic diagnostic codes according to the ICD-10-CM classification system. The main findings of this study are summarized in three key points:

1. The model was developed using a total of 28 variables selected from the initial 827 diagnostic features based on univariable statistical significance and absence of multicollinearity.
2. Internal performance in the derivation cohort was moderate. In the primary cross-validation analysis, predictor selection was repeated within each training fold and yielded a pooled out-of-fold ROC-AUC of 0.711, whereas the fixed 28-predictor specification produced a higher but more optimistic ROC-AUC of 0.738.
3. Patients classified by the fixed-predictor model as having reduced LVEF in the population-based cohort, exhibited a worse overall prognosis, with a higher risk of mortality and MACE compared with the group predicted to have preserved LVEF.

*Technical performance of the model:* A logistic regression model was chosen for its suitability to the available dataset and because the project scope prioritized the creation of an interpretable reference model before exploring more complex ML (Machine Learning) approaches.

The primary estimate of discrimination was obtained from a stratified 10-fold cross-validation framework in which predictor selection was repeated independently within each training fold. This approach resulted in a pooled out-of-fold ROC-AUC of 0.711 and incorporated all feature-selection steps within the validation process. The fixed 28-predictor specification achieved a higher ROC-AUC (0.738), reflecting the effect of selecting predictors before cross-validation. Despite this difference, both modelling strategies showed moderate discriminative ability.

Importantly, recall remained limited (≈0.30), indicating that most patients with measured LVEF < 50% were not identified at the selected classification threshold. Consequently, the model achieved high specificity but relatively low sensitivity. This trade-off illustrates the challenge of maximizing case detection while limiting false-positive classifications.

This performance is broadly consistent with previous attempts to infer LVEF from administrative or routinely collected data. For example, a recent large-scale claims-based study by Quentin et al. used a random forest approach and reported AUCs between 0.79 and 0.84 for different LVEF thresholds, but relied on a nationwide dataset with medication codes, hospitalization patterns and natriuretic peptide testing, variables not used in our study. Similarly, an ICU-based fuzzy-logic model derived from physiological monitoring data predicted severely depressed LVEF (≤25%) with an AUC of 0.71, outperforming its own logistic-regression benchmark (AUC 0.67). Compared with these studies, the performance of our model aligns with expectations given that it relied almost exclusively on diagnostic codes [26,27].

Among the final selected features, some contributed more strongly to the logistic model than others. In particular, the diagnose code I2109 (Acute ST-elevation MI with involvement of another anterior coronary artery) was the most influential, together with Killip-Kimbal classification. The next four influential diagnose codes followed closely the previous two in importance, showing similar metrics. The remaining variables exhibited a combined effect, suggesting that this model integrated multiple lower-weighted clinical diagnose factors. These predictors are clinically plausible, as they reflect established risk markers for LV dysfunction [28]. Notably, anterior STEMI and Killip class are themselves established markers of ACS severity, so the model may in part be capturing disease severity at presentation rather than independently reconstructing LVEF (see Limitations).

Because predictor selection was performed before cross-validation in the fixed 28-predictor specification, its performance estimates should be interpreted with caution. Repeating predictor selection within each training fold resulted in a lower ROC-AUC (0.711 versus 0.738) and provides the primary estimate of model discrimination. External validation in an independent cohort with reference LVEF measurements remains necessary.

In the additional pooled out-of-fold assessment, calibration-in-the-large and calibration slope estimates were close to their ideal values, although sensitivity remained limited at the 0.50 threshold. These results support the interpretation of the model as a population-level epidemiological tool for characterizing groups of patients with different model-estimated probabilities of reduced LVEF in settings where measured LVEF is unavailable, rather than as an individual diagnostic, prognostic, or rule-out tool.

*Clinical performance of the model:* After applying our prediction model to a large cohort of patients hospitalized for ACS, 8% of patients were classified as having reduced LVEF (<50%). Although the proportion of patients with reduced LVEF after ACS varies across published studies, our estimate is lower than, but broadly compatible with findings from studies conducted in similar sociodemographic settings, such as that of Bermejo et al., who reported a 16% prevalence of reduced LVEF among a cohort of 5064 patients hospitalized for ACS without concomitant HF [7].

Patients with a prLVEF exhibited a higher incidence of all-cause mortality during follow-up, with a HR of 2.45. This prognostic gradient is compatible with the well-established association between measured reduced LVEF and adverse outcomes after ACS reported in previous studies, which have found HRs for mortality ranging from 1.64 to 2.13 [29,30,31]. However, the prognostic analyses performed in the population-based cohort should not be interpreted as demonstrating the independent prognostic value of prLVEF beyond variables already incorporated into the prediction model. Several influential predictors, including anterior STEMI and Killip class, are well-established markers of ACS severity and myocardial injury. Therefore, the model likely captures, at least in part, the severity of the index ACS episode, which is biologically linked to the development of reduced LVEF. In addition, because diagnosis-level timestamps were unavailable in the analytical dataset, individual diagnostic codes could not be temporally distinguished as pre-existing conditions or in-hospital complications. Consequently, the model should be interpreted as identifying clinical profiles in which reduced LVEF is more likely to be present rather than reconstructing LVEF independently of these prognostic characteristics.

Patients with a prLVEF also demonstrated a poorer overall prognosis, with an increased incidence of MACE during follow-up (HR 1.64) and a higher risk of developing a terminal event (HR 2.47). Similarly, a recent meta-analysis including 40 studies of patients with ACS undergoing percutaneous coronary revascularization showed that reduced LVEF is an independent prognostic factor for the development of MACE in this population, with an OR of 1.86 (95% CI [1.71–2.03]; *p* < 0.0001) [32].

In an era of increasing availability of large-scale administrative and real-world healthcare data routinely collected and centralized by nationwide healthcare organizations, the ability to infer clinically meaningful parameters from diagnostic codes represents a major opportunity for clinical research. However, these administrative databases typically contain diagnoses and resource use, missing essential clinical information in the cardiovascular field, such as cardiac imaging data and measured LVEF. Models such as the one developed here may help address this limitation by enabling exploratory population-level epidemiological analyses in settings where reference LVEF measurements are unavailable. Their use should remain limited to this research context until externally validated against standardized LVEF measurements.

*Limitations*: This study has some inherent limitations that warrant clarification. (I) A logistic regression model was selected as a first approach due to its interpretability and suitability for clinical data. Further refinement of the model, including different methods for variable selection and algorithm comparison, will be necessary to improve its robustness and generalizability. (II) The model was developed using retrospective data from a single tertiary centre, which may limit its generalizability to other populations and healthcare settings. (III) The diagnoses of patients from the derivation cohort between 2009–2010 were originally coded in ICD-9-CM. Conversion to ICD-10-CM using internal mapping tables to align with current diagnostic coding was performed. Although the conversion tables were constructed to ensure the closest possible equivalences, ICD-9-CM has a more generic structure with less detail compared to ICD-10-CM, which provides richer information, specificity and a larger set of subcategories. It cannot be overlooked that a model trained directly on ICD-10-CM would likely achieve higher accuracy. (IV) Although the model demonstrated moderate discriminative ability, its relatively low sensitivity could lead to under-identification of patients with reduced LVEF, potentially impacting clinical applicability. At the 0.50 threshold, 320 of 454 patients with measured LVEF <50% were classified as negative. Therefore, the model should not be used to rule out reduced LVEF or to decide which individual patients should undergo echocardiography. (V) The model has not been validated in a cohort of patients with known LVEF values. This lack of external validation prevents definitive confirmation of the model’s accuracy and ability to estimate or classify LVEF in real-world clinical settings. Therefore, additional studies incorporating reference data obtained through standardized LVEF measurement techniques will be necessary to assess the model’s performance and applicability across diverse populations. (VI) Predictor screening and collinearity assessment were initially conducted before cross-validation when deriving the fixed 28-predictor specification subsequently applied to the population-based cohort. Repeating all predictor-selection steps independently within each training fold resulted in a lower ROC-AUC (0.711 versus 0.738) and provided the primary estimate of model discrimination. Nevertheless, neither approach replaces external validation in an independent cohort with measured LVEF. (VII) Calibration was assessed internally using pooled out-of-fold predictions. External calibration could not be assessed because reference LVEF measurements were unavailable in the population-based cohort. (VIII) Although the predictor window was restricted to information available up to discharge from the index ACS hospitalization, the analytical binary matrix did not retain diagnosis-specific timestamps or diagnostic rank. Individual codes could therefore not be retrospectively classified as pre-existing diagnoses, diagnoses newly documented during admission, or in-hospital complications. Consequently, the precise temporal relationship between individual diagnostic codes and the echocardiographic assessment of LVEF could not be established. Some predictors may therefore reflect acute ACS severity or complications occurring during hospitalization rather than pre-admission patient characteristics. (IX) The model-predicted LVEF classification integrates 26 diagnostic codes together with age and Killip class into a single composite score. Age and Killip class are themselves established prognostic markers after ACS; the prognostic gradient observed between predicted groups therefore reflects, at least in part, the combined severity captured by all model inputs, rather than an effect attributable to predicted LVEF status in isolation. Dis-entangling the incremental contribution of the composite classification from its individual components was beyond the scope of the present analysis. The observed gradient is nonetheless consistent with the well-established prognostic role of reduced LVEF, Killip class and age individually, and is consistent with differences in observed outcomes between the model-defined groups in a population where LVEF itself is not directly observable. (X) The primary intended use of this model is population-level characterization of LVEF status in administrative databases where measured LVEF is unavailable, rather than individual patient screening or clinical decision-making.

## 5. Conclusions

This study explored the feasibility of estimating the probability of reduced LVEF after ACS from ICD-10-CM diagnostic codes and two clinical variables. The predictive model was built on logistic regression. In the derivation cohort, the primary estimate of model discrimination, obtained using a cross-validation framework that repeated predictor selection independently within each training fold, showed moderate performance (ROC-AUC 0.711). The fixed 28-predictor model subsequently applied to the population-based cohort achieved a higher ROC-AUC (0.738).

At the 0.50 threshold, the model missed 320 of 454 patients with measured reduced LVEF in the derivation cohort. When applied to a large population-based cohort with unknown LVEF, the model classified individuals into two model-defined groups that differed in clinical characteristics and observed outcomes. Importantly, patients classified as having prLVEF exhibited significantly worse clinical outcomes, including higher mortality and cardiovascular event rates, compared to those predicted to have preserved LVEF.

Further refinement of the model, such as optimizing variable selection and performing external validation, will be necessary to improve its robustness and generalizability. These findings support further evaluation of the model for exploratory population-level epidemiological and prognostic analyses, but not as a stand-alone diagnostic test, a rule-out tool, or an individual clinical decision rule. External validation against measured LVEF in an independent population is required before diagnostic performance or clinical applicability can be established.

## Figures and Tables

**Table 1:** Table describing baseline characteristics of the derivation cohort (1596 patients) according to LVEF value. Patients meeting the administrative HF exclusion criterion were excluded from this cohort. AF: atrial fibrillation; CABG: coronary artery bypass grafting; CKD: chronic kidney disease; COPD: Chronic obstructive pulmonary disease; DLP: dyslipidaemia; HTN; hypertension; MI: Myocardial infarction; PAD: peripheral arterial disease; STEMI: ST-elevation MI.

	LVEF ≥ 50%	LVEF < 50%	*p* Value
Patients (*n*)	1142	454	
Female sex (*n* (%))	239 (20.9)	89 (19.6)	0.555
Age (mean (SD))	61.12 (12.2)	63.12 (13)	0.005
Age > 75 years (*n* (%))	160 (14.0)	92 (20.3)	0.002
MI location (*n* (%))			<0.001
Anterior	396 (34.7)	272 (59.9)	
Inferior	581 (50.9)	119 (26.2)	
Lateral	62 (5.4)	21 (4.6)	
Posterior	20 (1.8)	12 (2.6)	
Other	83 (7.3)	30 (6.6)	
Killip class (*n* (%))			<0.001
I	1077 (94.3)	333 (73.3)	
II	49 (4.3)	82 (18.1)	
III	10 (0.9)	22 (4.8)	
IV	6 (0.5)	17 (3.7)	
Killip class > I (*n* (%))	65 (5.7)	121 (26.7)	<0.001
Smoke habit (*n* (%))			0.198
Non-smoker	296 (25.9)	120 (26.4)	
Former	317 (27.8)	144 (31.7)	
Active	529 (46.3)	190 (41.9)	
DM (*n* (%))	289 (25.3)	162 (35.7)	<0.001
HTN (*n* (%))	653 (57.2)	256 (56.4)	0.773
DLP (*n* (%))	669 (58.6)	271 (59.7)	0.684
PAD (*n* (%))	116 (10.2)	49 (10.8)	0.707
CKD (*n* (%))	63 (5.5)	33 (7.3)	0.184
Previous MI (*n* (%))	113 (9.9)	75 (16.5)	<0.001
Previous CABG (*n* (%))	23 (2.0)	15 (3.3)	0.127
Previous AF (*n* (%))	32 (2.8)	25 (5.5)	0.009
COPD (*n* (%))	146 (12.8)	73 (16.1)	0.084
Anaemia (*n* (%))	39 (3.4)	32 (7.0)	0.001
STEMI (*n* (%))	742 (65.0)	333 (73.3)	0.001
Previous Stroke (*n* (%))	56 (4.9)	39 (8.6)	0.005

**Table 2:** ICD-10-CM codes and meaning.

Code	Description
Age > 75	Age greater than 75
B9561	Infection by methicillin-sensitive Staphylococcus aureus as a cause of diseases classified under another concept
B958	Unspecified staphylococcus as a cause of diseases classified under another concept
E118	Diabetes mellitus type 2 with unspecified complications
E119	Diabetes mellitus type 2 without complications
E669	Obesity unspecified
F070	Alteration of personality due to a known physiological condition
F1020	Alcohol dependence without complications
I129	Hypertensive chronic renal disease with chronic renal disease in stages 1 to 4 or with unspecified chronic renal disease
I2109	Acute ST elevation myocardial infarction (STEMI) with involvement of another anterior coronary artery
I2111	Acute ST elevation myocardial infarction (STEMI) with right coronary artery involvement
I2119	Acute ST elevation myocardial infarction (STEMI) with involvement of another inferior coronary artery
I214	Acute non-ST elevation myocardial infarction (NSTEMI)
I428	Other cardiomyopathies
I447	Left bundle branch block unspecified
I469	Cardiac arrest unspecified cause
I472	Ventricular tachycardia
I4891	Atrial fibrillation not specified
I519	Heart disease unspecified
I700	Aortic atherosclerosis
J690	Pneumonitis due to inhalation of food and vomiting
J9600	Acute respiratory failure not specified if with hypoxia or hypercapnia
Killip > 1	Killip–Kimball classification value greater than 1
M10	Gout
M109	Gout unspecified
R6520	Severe sepsis without septic shock
Y848	Other medical procedures as cause of abnormal patient reaction or subsequent complication without mention of an adverse event at the time of the procedure
Z79899	Other long-term (current) drug therapy

**Table 3:** Baseline characteristics of the patients included in the stratification potential evaluation cohort (overall/LVEF < 50%/LVEF ≥ 50%). LVEF: left ventricular ejection fraction.

	Overall (*N* = 83,357)	ppLVEF (Predicted ≥50%) (*N* = 76,721)	prLVEF (Predicted <50%) (*N* = 6636)	*p* Value
Gender Female (*n* (%))	23,814 (28.6%)	21,682 (28.3%)	2132 (32.1%)	<0.001
Age (mean (SD))	66.8 (13.4)	66.3 (13.2)	72.7 (13.6)	<0.001
User of the public healthcare system (*n* (%))	79,881 (95.8%)	73,590 (95.9%)	6291 (94.8%)	<0.001
Morbidity burden GMA (mean (SD))	16.8 (10.6)	16.5 (10.4)	20.3 (12.4)	<0.001
Diabetes mellitus (*n* (%))	28,781 (34.5%)	25,494 (33.2%)	3287 (49.5%)	<0.001
Hypertension (*n* (%))	59,888 (71.8%)	54,805 (71.4%)	5083 (76.6%)	<0.001
Hyperlipidaemia (*n* (%))	55,980 (67.2%)	51,828 (67.6%)	4152 (62.6%)	<0.001
Obesity (*n* (%))	25,258 (30.3%)	23,579 (30.7%)	1679 (25.3%)	<0.001
Smoking habit (*n* (%))	49,327 (59.2%)	45,994 (59.9%)	3333 (50.2%)	<0.001
Chronic kidney disease (*n* (%))	13,877 (16.6%)	11,963 (15.6%)	1914 (28.8%)	<0.001
Killip class (*n* (%))				0.000
Class I	75,216 (90.2%)	72,431 (94.4%)	2785 (42.0%)	
Class II–III	6802 (8.16%)	3997 (5.21%)	2805 (42.3%)	
Class IV	1339 (1.61%)	293 (0.38%)	1046 (15.8%)	

**Table 4:** Total number of events and incidence rates according to exposure status in the overall cohort and stratified by model-predicted left ventricular ejection fraction (LVEF < 50% vs. ≥50%). New CV intervention = PCI or CABG performed during follow-up. Results are presented as event counts and incidence rates per 100 person-years. CV: cardiovascular; LVEF: left ventricular ejection fraction; HF: heart failure; MCS: mechanical circulatory support; PCI: percutaneous coronary intervention; CABG: coronary artery bypass grafting; ACS: acute coronary syndrome; MI: myocardial infarction; TIA: transient ischemic attack; AF: atrial fibrillation; MACE: major adverse cardiovascular events; HTx: heart transplantation.

	Overall Event (Incident Rate × 100)	ppLVEF (Predicted ≥50%) Event (Incident Rate × 100)	prLVEF (Predicted <50%) Event (Incident Rate × 100)
Incident HF	19,417 (5.2)	16,117 (4.7)	3300 (12.5)
All-cause death	18,080 (4.9)	15,221 (4.4)	2859 (10.9)
MCS implant	65 (0.0)	47 (0.0)	18 (0.1)
Heart transplant	65 (0.0)	36 (0.0)	29 (0.1)
New PCI intervention	26,349 (7.1)	24,509 (7.1)	1840 (7.0)
New CABG intervention	6797 (1.8)	6442 (1.9)	355 (1.3)
New CV intervention	33,146 (9.0)	30,951 (9.0)	2195 (8.3)
Recurrent ACS	24,059 (6.5)	22,181 (6.4)	1878 (7.1)
Recurrent MI	12,473 (3.4)	11,242 (3.3)	1231 (4.7)
Stroke or TIA	5146 (1.4)	4663 (1.4)	483 (1.8)
New AF/flutter	7341 (2.0)	6645 (1.9)	696 (2.6)
4-point MACE	80,431 (21.7)	73,016 (21.2)	7415 (28.2)
Terminal event (death, Htx, MCS)	18,210 (4.9)	15,304 (4.4)	2906 (11.0)

**Table 5:** Cumulative probability of first event and hazard ratios derived from time-to-first-event analyses according to model-predicted LVEF group. Event counts correspond to patients experiencing a first event during follow-up. Recurrent events occurring in the same patient are not counted more than once.

	N with Events at 10 Years	% Probability of Event (95% CI)			HR (95% CI)	Signification
		2 Years	5 Years	10 Years		
All-cause death						
Predicted LVEF ≥ 50	15,221	8.8% (8.6–9.1%)	19% (19–19%)	37% (36–37%)	–	–
Predicted LVEF < 50	2859	24% (23–25%)	41% (39–42%)	61% (59–63%)	2.45 (2.35–2.55)	<0.001
MCS implant						
Predicted LVEF ≥ 50	47	0.04% (0.03–0.06%)	0.07% (0.05–0.09%)	0.09% (0.06–0.13%)	–	–
Predicted LVEF < 50	18	0.22% (0.13–0.36%)	0.26% (0.15–0.41%)	0.36% (0.20–0.61%)	4.20 (2.43–7.26)	<0.001
Heart transplant						
Predicted LVEF ≥ 50	36	0.03% (0.02–0.04%)	0.05% (0.03–0.07%)	0.08% (0.05–0.11%)	–	–
Predicted LVEF < 50	29	0.28% (0.17–0.44%)	0.46% (0.31–0.67%)	0.55% (0.37–0.79%)	8.70 (5.33–14.2)	<0.001
New PCI						
Predicted LVEF ≥ 50	10,699	11% (11–12%)	15% (14–15%)	18% (18–18%)	–	–
Predicted LVEF < 50	801	9.8% (9.1–11%)	12% (11–13%)	14% (13–16%)	0.82 (0.77–0.88)	<0.001
New CABG						
Predicted LVEF ≥ 50	3366	4.1% (3.9–4.2%)	4.5% (4.3–4.6%)	5.1% (4.9–5.3%)	–	–
Predicted LVEF < 50	197	2.7% (2.3–3.1%)	3.0% (2.6–3.5%)	3.3% (2.9–3.9%)	0.66 (0.57–0.76)	<0.001
New CV intervention						
Predicted LVEF ≥ 50	13,552	15% (15–15%)	18% (18–19%)	22% (22–22%)	–	–
Predicted LVEF < 50	970	12% (11–13%)	15% (14–16%)	17% (16–18%)	0.78 (0.73–0.84)	<0.001
Recurrent ACS						
Predicted LVEF ≥ 50	13,622	13% (13–13%)	18% (18–19%)	24% (24–25%)	–	–
Predicted LVEF < 50	1211	14% (13–15%)	18% (18–19%)	22% (21–23%)	0.99 (0.93–1.05)	0.7
Recurrent MI						
Predicted LVEF ≥ 50	7710	7.1% (6.9–7.3%)	10% (10–11%)	14% (14–15%)	–	–
Predicted LVEF < 50	877	10% (9.6–11%)	13% (12–14%)	16% (15–17%)	1.28 (1.19–1.37)	<0.001
Stroke or TIA						
Predicted LVEF ≥ 50	3254	2.0% (1.9–2.2%)	4.1% (4.1–4.4%)	7.5% (7.2–7.8%)	–	–
Predicted LVEF < 50	389	3.3% (2.9–3.8%)	5.6% (5.1–6.3%)	8.1% (7.2–9.0%)	1.29 (1.16–1.43)	<0.001
New AF/Flutter						
Predicted LVEF ≥ 50	6645	4.4% (4.2–4.5%)	8.5% (8.3–8.7%)	15% (14–15%)	–	–
Predicted LVEF < 50	696	5.6% (5.1–6.2%)	9.8% (9.0–11%)	16% (15–17%)	1.13 (1.05–1.23)	0.002
4-point MACE						
Predicted LVEF ≥ 50	31,249	31% (31–32%)	43% (42–43%)	56% (55–56%)	–	–
Predicted LVEF < 50	3826	48% (47–49%)	59% (58–61%)	69% (67–71%)	1.64 (1.59–1.70)	<0.001
Terminal event						
Predicted LVEF ≥ 50	15,266	8.9% (8.7–9.1%)	19% (19–19%)	37% (36–37%)	–	–
Predicted LVEF < 50	2885	25% (23–26%)	41% (40–42%)	62% (60–64%)	2.47 (2.37–2.57)	<0.001

## Data Availability

The datasets analysed during the current study are not publicly available due to ethical and legal restrictions governing the use of healthcare data and patient confidentiality.

## Supplementary Materials

The following supporting information can be downloaded at https://www.mdpi.com/article/10.3390/jcdd13100524/s1, Table S1. The 30 most frequent ICD-10-CM diagnostic codes among the 1596 patients. Table S2. VIF values for every variable that will be introduced in the logistic regression model. Table S3. Frequency of predictor selection across the ten training folds in the primary internal validation analysis. Table S4. Fold-level results from the primary internal validation analysis. Table S5. Performance and calibration metrics from the primary internal validation analysis based on pooled out-of-fold predictions. Values are reported as estimate (95% bootstrap confidence interval). Table S6. Fixed 28-predictor cross-validation summary and additional pooled out-of-fold assessment. The original results are reported as mean ± SD across the 10 cross-validation folds. The additional pooled OOF estimates were calculated by retaining one held-out predicted probability per participant. Figure S1. Pooled out-of-fold ROC curve from the leakage-aware primary internal validation analysis.

## Abbreviations

The following abbreviations are used in this manuscript:

ACS	Acute Coronary Syndrome
AUC	Area Under the Curve
CI	Confidence Intervals
CV	Cross-Validation
HF	Heart Failure
HR	Hazard Ratio
ICD-10-CM	International Classification of Diseases, Tenth Revision, Clinical Modification
ICD-9-CM	International Classification of Diseases, 9th Revision, Clinical Modification
LVEF	Left Ventricular Ejection Fraction
MACE	Major Adverse Cardiovascular Events
MI	Myocardial Infarction
ML	Machine Learning
OR	Odds Ratio
pLVEF	Preserved Left Ventricular Ejection Fraction
ppLVEF	Predicted Preserved Left Ventricular Ejection Fraction
prLVEF	Predicted Reduced Left Ventricular Ejection Fraction
ROC	Receiver Operating Characteristic
SD	Standard Deviation
SHAP	Shapley Additive Explanations
TIA	Transient Ischemic Attack
VIFs	Variance Inflation Factors
