# Supplementary material for: Development and Population-Based Prognostic Utility Assessment of an ICD-10-CM-Based Prediction Model to Identify Left Ventricular Systolic Dysfunction: Discrimination Performance and Population-Based Evaluation in 83,357 Patients with Acute Coronary Syndrome
Source: J Cardiovasc Dev Dis. 2026 Oct 17;13(10):524. doi: 10.3390/jcdd13100524 (PMC13705362; doi:10.3390/jcdd13100524)
Supplement: Supplementary file 1 [file jcdd-13-00524-s001.zip › jcdd-4431547-supplementary.pdf]

Supplementary Materials

Table S1. The 30 most frequent ICD-10-CM diagnostic codes among the 1596 patients.

ICD-10-CM diagnostic codes	Frequency
E785	769
I10	703
F17200	635
Z87891	329
E119	303
Z8249	241
Z7982	171
F1010	165
E669	125
I739	120
Z9861	108
B953	91
I4891	86
I4510	86
Z794	78
N400	74
J449	61
Z7902	59
N179	57
Z961	53
K449	53
I519	52
Z9849	51
I129	51
I358	50
I442	50
Z880	49
Z8711	49
Z7901	44
J42	43

Table S2. VIF values for every variable that will be introduced in the logistic regression model.

Variable	VIF
E118	1.012386
M109	1.020771
F070	1.029164
B9561	1.033659
B958	1.036823
I519	1.044361
I447	1.047178
I129	1.076693
I700	1.097582
E669	1.097987
J9600	1.106518
R6520	1.112503
M10	1.128649
I472	1.135502
Z79899	1.137648
I4891	1.145825
I2119	1.148842
Y848	1.153958
J690	1.155767
F1020	1.161023
I428	1.161936
I2111	1.186327
I214	1.217383
I469	1.220415
I2109	1.249019
E119	1.270958
age75	1.302515
Killip Class > 1	1.329672

Table S3. Frequency of predictor selection across the 10 training folds in the primary internal validation analysis.

Feature	n_folds_selected	selection_frequency
B9561	10	1.0
B958	10	1.0
E118	10	1.0
F070	10	1.0
F1020	10	1.0
I2109	10	1.0
I2111	10	1.0
I2119	10	1.0
I214	10	1.0
I428	10	1.0
I447	10	1.0
I472	10	1.0
I4891	10	1.0
I519	10	1.0

I700	10	1.0
J690	10	1.0
M109	10	1.0
R6520	10	1.0
Z79899	10	1.0
age75	10	1.0
killipmajor1	10	1.0

Table S4. Fold-level results from the primary internal validation analysis.

Fold	Diagnostic codes selected	Total predictors	ROC-AUC	Accuracy	Sensitivity	Specificity	Maximum VIF	F1-score
1	43	45	0.684	0.769	0.378	0.922	3.797	0.479
2	42	44	0.781	0.738	0.222	0.939	2.299	0.323
3	46	48	0.687	0.756	0.217	0.974	3.945	0.339
4	46	48	0.648	0.744	0.283	0.930	2.177	0.388
5	46	48	0.743	0.769	0.326	0.947	3.794	0.448
6	41	43	0.702	0.744	0.304	0.921	4.046	0.406
7	42	44	0.704	0.755	0.289	0.939	2.185	0.400
8	44	46	0.714	0.761	0.289	0.947	4.743	0.406
9	36	38	0.725	0.774	0.333	0.947	3.793	0.455
10	44	46	0.764	0.748	0.244	0.947	4.078	0.355

Table S5. Performance and calibration metrics from the primary internal validation analysis based on pooled out-of-fold predictions. Values are reported as estimate (95% bootstrap confidence interval).

ROC-AUC	Accuracy	Sensitivity	Specificity	Calibration-in-the-large	Calibration slope	Brier score
0.711 (0.680– 0.741)	0.756 (0.735–0.776)	0.289 (0.248–0.330)	0.941 (0.928–0.955)	–0.097 (–0.244–0.066)	0.874 (0.749–1.005)	0.172 (0.161–0.183)

Table S6. Fixed 28-predictor cross-validation summary and additional pooled out-of-fold assessment. The original results are reported as mean ± SD across the 10 cross-validation folds. The additional pooled OOF estimates were calculated by retaining one held-out predicted probability per participant.

**Metric**	**Summary 10-fold CV report in manuscript***	**Additional pooled OOF assessment from 10-fold CV**
Validation procedure	Mean across 10 stratified test folds	One held-out predicted probability per patient, pooled across the 10 folds
N	1596	1596
Outcome prevalence, LVEF <50%	28.5%	28.5%
ROC-AUC	0.738 ± 0.040	0.732 (95% CI 0.702–0.761)
PR-AUC	Not reported	0.578 (95% CI 0.532–0.627)
Accuracy	0.764 ± 0.015	0.765 (95% CI 0.744–0.785)
Precision / PPV	0.713 ± 0.080	0.709 (95% CI 0.644–0.774)
Recall / sensitivity	0.295 ± 0.029	0.295 (95% CI 0.254–0.337)
Specificity	Not reported	0.952 (95% CI 0.939–0.964)
NPV	Not reported	0.773 (95% CI 0.751–0.793)
F1-score	0.415 ± 0.028	0.417 (95% CI 0.369–0.463)
Brier score	Not reported	0.166 (95% CI 0.155–0.177)
Null-model Brier score†	Not reported	0.204
Calibration-in-the-large	Not reported	−0.010 (95% CI −0.181 to 0.158)
Calibration slope	Not reported	0.976 (95% CI 0.852–1.111)
True positives, cutoff 0.50	Not reported	134
False negatives, cutoff 0.50	Not reported	320
True negatives, cutoff 0.50	Not reported	1087
False positives, cutoff 0.50	Not reported	55

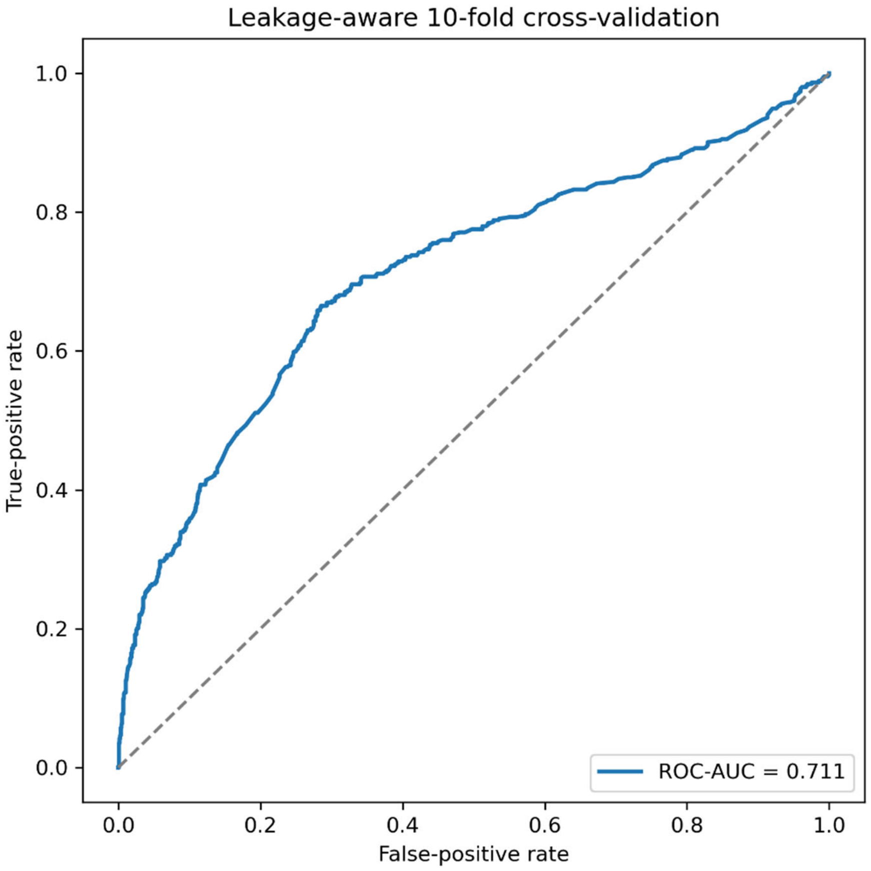

Figure S1. Pooled out-of-fold ROC curve from the leakage-aware primary internal validation analysis.
